# Mitochondrial pyruvate carrier inhibitors improve metabolic parameters in diet-induced obese mice

**DOI:** 10.1016/j.jbc.2021.101554

**Published:** 2021-12-30

**Authors:** Wesley T. Hodges, Chaowapong Jarasvaraparn, Daniel Ferguson, Kristine Griffett, Lauren E. Gill, Yana Chen, Ma. Xenia G. Ilagan, Lamees Hegazy, Bahaa Elgendy, Kevin Cho, Gary J. Patti, Kyle S. McCommis, Brian N. Finck

**Affiliations:** 1 Department of Medicine, Washington University School of Medicine, St. Louis, Missouri, USA; 2 Center for Clinical Pharmacology, Washington University School of Medicine and University of Health Sciences & Pharmacy, St. Louis, Missouri, USA; 3 Department of Pharmaceutical and Administrative Sciences, University of Health Sciences and Pharmacy, St. Louis, Missouri, USA; 4 Department of Biochemistry and Molecular Biology, Saint Louis University School of Medicine, St. Louis, Missouri, USA

**Keywords:** mitochondrial metabolism, pyruvate, diabetes, gluconeogenesis, metabolic disease, BRET, bioluminescence resonance energy transfer, DG, deoxyglucose, DIO, diet-induced obese, DMEM, Dulbecco's modified Eagle's medium, DMSO, dimethyl sulfoxide, FBS, fetal bovine serum, GTT, glucose tolerance test, HBSS, Hank's Buffered Saline Solution, HF, high-fat, ITT, insulin tolerance test, LF, low-fat, MPC, mitochondrial pyruvate carrier, OCR, oxygen consumption rate, PDE, phosphodiesterase, PPARγ, peroxisome proliferator–activated receptor γ, RESPYR, reporter sensitive to pyruvate, TZD, thiazolidinedione

## Abstract

The mitochondrial pyruvate carrier (MPC) is an inner mitochondrial membrane complex that plays a critical role in intermediary metabolism. Inhibition of the MPC, especially in liver, may have efficacy for treating type 2 diabetes mellitus. Herein, we examined the antidiabetic effects of zaprinast and 7ACC2, small molecules which have been reported to act as MPC inhibitors. Both compounds activated a bioluminescence resonance energy transfer–based MPC reporter assay (reporter sensitive to pyruvate) and potently inhibited pyruvate-mediated respiration in isolated mitochondria. Furthermore, zaprinast and 7ACC2 acutely improved glucose tolerance in diet-induced obese mice *in vivo*. Although some findings were suggestive of improved insulin sensitivity, hyperinsulinemic–euglycemic clamp studies did not detect enhanced insulin action in response to 7ACC2 treatment. Rather, our data suggest acute glucose-lowering effects of MPC inhibition may be due to suppressed hepatic gluconeogenesis. Finally, we used reporter sensitive to pyruvate to screen a chemical library of drugs and identified 35 potentially novel MPC modulators. Using available evidence, we generated a pharmacophore model to prioritize which hits to pursue. Our analysis revealed carsalam and six quinolone antibiotics, as well as 7ACC1, share a common pharmacophore with 7ACC2. We validated that these compounds are novel inhibitors of the MPC and suppress hepatocyte glucose production and demonstrated that one quinolone (nalidixic acid) improved glucose tolerance in obese mice. In conclusion, these data demonstrate the feasibility of therapeutic targeting of the MPC for treating diabetes and provide scaffolds that can be used to develop potent and novel classes of MPC inhibitors.

Obesity is associated with an increased risk of several chronic and progressive diseases, including insulin resistance and type 2 diabetes mellitus, which constitute a significant public health burden. Clinically approved drugs for type 2 diabetes employ a number of approaches for lowering blood glucose including augmenting the release of insulin by pancreatic beta cells, decreasing reabsorption of glucose by the kidneys, suppressing the production of glucose by the liver, or enhancing the sensitivity of target tissues to the effects of insulin. The thiazolidinedione (TZD) class of drugs, including rosiglitazone and pioglitazone, act as insulin sensitizers and are agonists for a nuclear receptor transcription factor, the peroxisome proliferator–activated receptor γ (PPARγ) (1), which mediates many of their beneficial effects. However, TZDs are known to interact with additional molecular targets and can affect metabolism by mechanisms other than transcription regulation (2, 3, 4). Indeed, TZDs rapidly suppress hepatic glucose production (5, 6), and recent work has suggested that TZDs with very limited activation of PPARγ (PPARγ-sparing TZDs) also have beneficial metabolic effects (7, 8, 9, 10, 11). This PPARγ-independent pharmacology has been linked to the ability of these compounds to interact with the mitochondrial pyruvate carrier (MPC) complex (3, 4).

The MPC is composed of two proteins, MPC1 and MPC2, in a heterodimeric complex that mediates the transport of pyruvate across the inner mitochondrial membrane into the mitochondrial matrix (12, 13). This is an important and rate-limiting step in intermediary metabolism. Both MPC1 and MPC2 are required for pyruvate transport and complex stability, and thus, the deletion of one MPC protein essentially results in a double knockout and complete loss of pyruvate transport activity (14). Constitutive deletion of MPC1 or MPC2 in mice results in lethality at an early embryonic stage (14, 15). However, conditional deletion of either MPC proteins in hepatocytes is well tolerated and results in protection from diabetes, liver injury, and other high-fat (HF) diet–induced metabolic derangements (9, 16, 17, 18). This fits well with the accumulating evidence that TZDs targeting the MPC act in an inhibitory manner to suppress the flow of pyruvate into mitochondrial metabolic pathways. While the metabolic benefit of interrupting normal pyruvate use in glucose-consuming tissues, such as skeletal muscle, may seem counterintuitive, impairing mitochondrial pyruvate flux in the liver is beneficial in certain disease states by reducing gluconeogenesis (16, 17, 18). Moreover, inhibition of the MPC also stimulates fat oxidation and compensatory use of amino acids by the liver (7, 16), brown adipose tissue (19, 20), and skeletal muscle (21).

Herein, we tested the hypothesis that small-molecule inhibitors of the MPC can improve metabolic phenotypes in the setting of obesity in mice. First, we confirmed previous reports that zaprinast (22), a phosphodiesterase (PDE) inhibitor, and 7ACC2 (23), originally believed to be a plasma membrane monocarboxylate transporter inhibitor, directly interact with the MPC using a bioluminescence resonance energy transfer (BRET)–based MPC conformation sensor (reporter sensitive to pyruvate [RESPYR]) (24). We then demonstrated that treatment with either compound acutely improved glucose tolerance in diet-induced obese (DIO) mice. Although some end points (insulin tolerance test [ITT] and insulin signaling measures) were suggestive of improved insulin sensitivity, hyperinsulinemic–euglycemic clamp studies did not detect enhanced insulin action in response to 7ACC2 treatment. Rather, our data suggest that acute metabolic improvements with the novel MPC inhibitors may be due to suppressed hepatic gluconeogenesis. Next, we used the RESPYR system to conduct a limited screen of potential MPC-interacting compounds in a 1600 compound chemical library of known drugs. This unbiased approach identified several known MPC inhibitors (zaprinast, pioglitazone, and rosiglitazone), as well as a variety of novel inhibitors of the MPC. To prioritize the novel MPC inhibitors from our screen for further investigation, we developed a pharmacophore model using the structure of the very potent MPC inhibitor, 7ACC2. Based on this model, we prioritized carsalam and six quinolone antibiotics that were hits in the screen and demonstrated that these compounds are novel inhibitors of the MPC. Collectively, these data validate a novel pharmacophore model for inhibiting the MPC and demonstrate the feasibility of therapeutic inhibition of the MPC for treating diabetes.

## Results

### Zaprinast and 7ACC2 are potent inhibitors of the MPC

**Figure 1:**
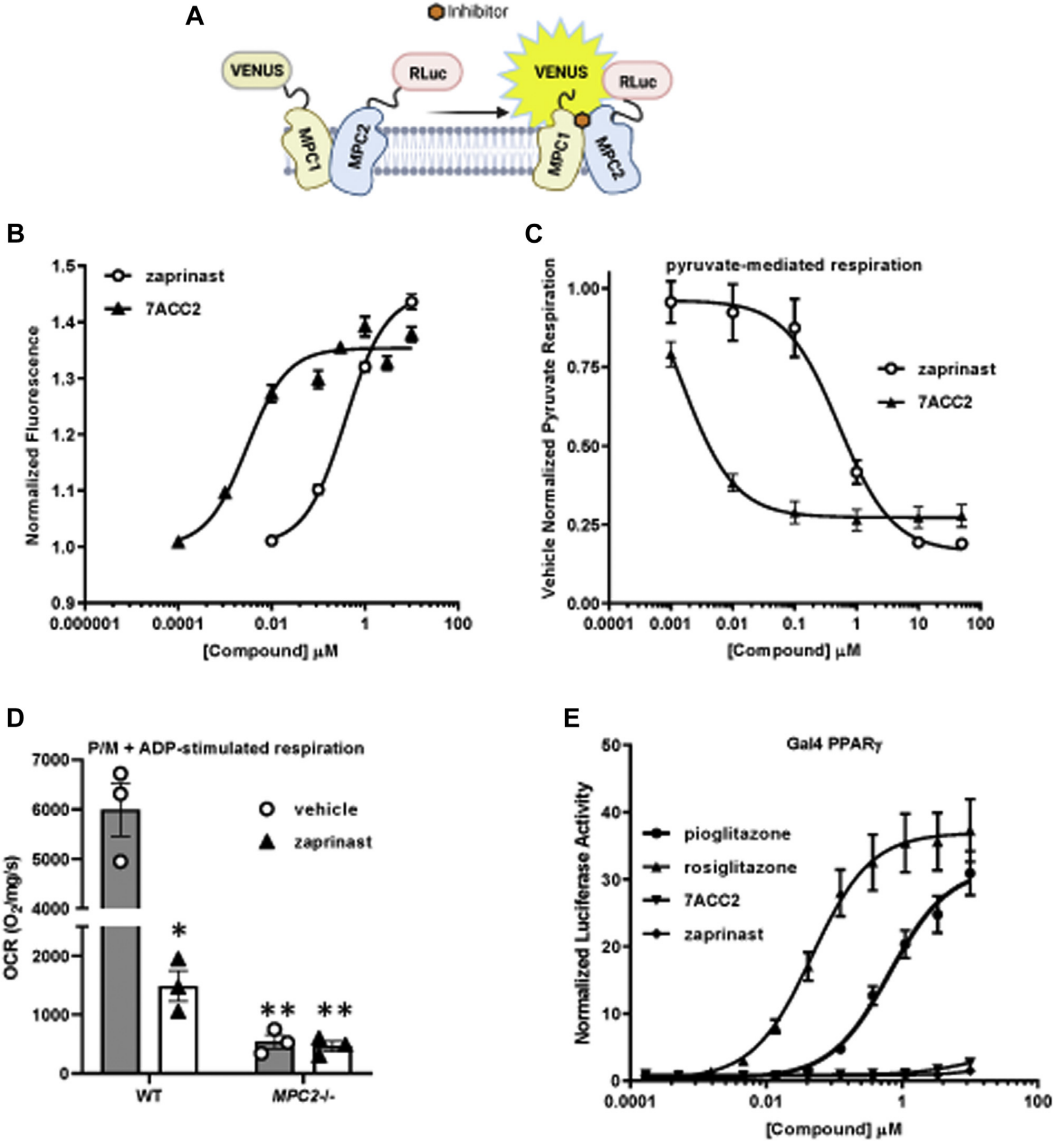
**Zaprinast and 7ACC2 are MPC inhibitors that do not activate PPARγ.***A*, the schematic depicts the BRET-based MPC biosensor (RESPYR) system with MPC1–Venus and MPC2-RLuc fusion proteins in the absence (*left*) or presence (*right*) of MPC inhibitors. Created with BioRender.com. *B*, dose–response effects of zaprinast or 7ACC2 in a RESPYR assay. Values are presented as mean ± standard error of the mean. n = 5 per group. *C*, pyruvate-stimulated mitochondrial respiration with increasing doses of zaprinast or 7ACC2. *D*, the effects of zaprinast on mitochondrial pyruvate metabolism require MPC. The effects of vehicle or zaprinast on pyruvate-stimulated respiration by cardiac mitochondria from WT or cardiac-specific MPC2-knockout mice are shown. *E*, zaprinast and 7ACC2 do not activate PPARγ. The effects of zaprinast, 7ACC2, rosiglitazone, and pioglitazone on the activity of a Gal4-PPARγ–driven luciferase reporter are shown. ∗*p* < 0.01 *versus* WT vehicle and *MPC2−/−,* ∗∗*p* < 0.01 *versus* all other groups. BRET, bioluminescence resonance energy transfer; MPC, mitochondrial pyruvate carrier; MPP, mitochondrial pyruvate carrier; OCR, oxygen consumption rate; PPARγ, peroxisome proliferator–activated receptor γ; RESPYR, reporter sensitive to pyruvate.

A BRET-based MPC activity assay (RESPYR) (24) has been shown to be sensitive to pharmacologic inhibitors of the MPC, including several TZDs and UK-5099 (8, 9). We have shown that MPC inhibitors induce a strong increase in BRET signal likely due to MPC complex conformational changes in response to engagement of the pyruvate binding site (Fig. 1*A*) (8, 9). Zaprinast, which has previously been reported to be an MPC inhibitor (22), activated RESPYR activity in a dose-dependent manner (Fig. 1*B*). Zaprinast was originally studied as a PDE inhibitor and was optimized to develop the PDE5 inhibitor, sildenafil. Sildenafil, tadalafil, and vardenafil did not activate RESPYR activity (Fig. S1*A*). A recent publication by Corbet *et al.* (23) suggested that 7ACC2, which was previously thought to be an inhibitor of cellular lactate import but not efflux, is actually an inhibitor of the MPC. We also confirmed that 7ACC2 was very potent at stimulating BRET activity in RESPYR analyses (Fig. 1*B*) demonstrating a direct interaction.

Zaprinast directly inhibited pyruvate-stimulated respiration in isolated mitochondria with an IC_50_ consistent with its ability to increase RESPYR signal (Fig. 1*C*). Consistent with the idea that zaprinast was affecting MPC independent of its effects on PDE activity, other PDE5 inhibitors did not inhibit pyruvate-stimulated respiration (Fig. S1*B*). By using mitochondria isolated from hearts of mice with cardiac-specific deletion of MPC2 (25), we confirmed that zaprinast only inhibited pyruvate-mediated respiration in mitochondria that expressed MPC (Fig. 1*D*). 7ACC2 also inhibited mitochondrial respiration in a dose-dependent manner when pyruvate was provided as metabolic substrate and, consistent with RESPYR dose–response curves, was more potent compared to zaprinast (Fig. 1*C*). Finally, given that other known MPC inhibitors also activate PPARγ, we confirmed, using a Gal4-PPARγ luciferase reporter assay, that 7ACC2 and zaprinast did not activate this nuclear receptor (Fig. 1*E*). Collectively, these data are consistent with a direct inhibitor effect of these compounds on MPC activity.

### Zaprinast and 7ACC2 improve glucose tolerance in DIO mice

**Figure 2:**
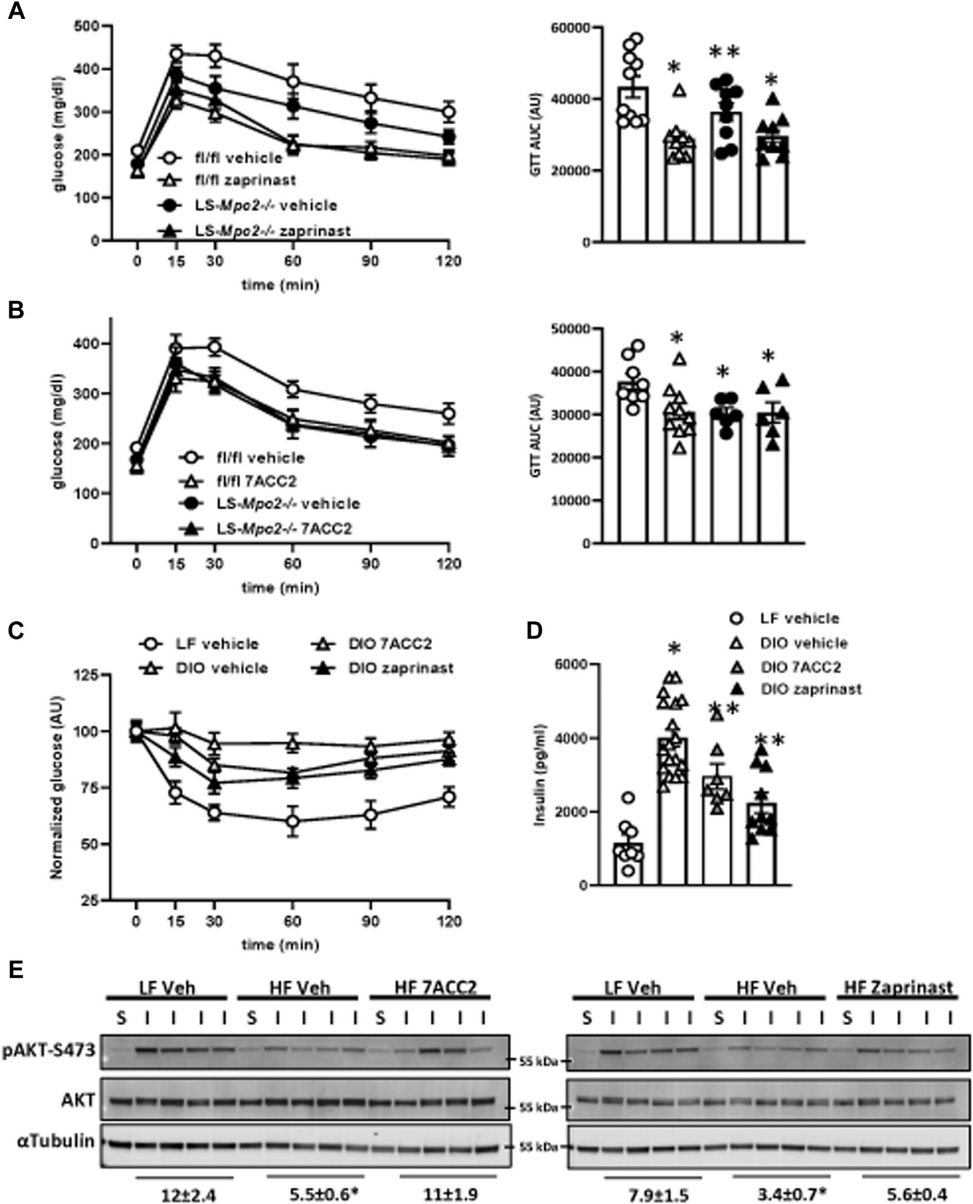
**Zaprinast and 7ACC2 improve glucose tolerance in DIO mice.***A* and *B*, WT and LS-Mpc2−/− mice were fed a high-fat (HF) diet for 12 weeks and then administered a single dose of zaprinast (*A*) or 7ACC2 (*B*) or vehicle control. Glucose tolerance was then assessed 16 h later after an overnight fast. ∗*p* < 0.05 compared to WT vehicle. ∗∗*p* < 0.05 compared to WT vehicle and all zaprinast-treated mice. *C*, insulin tolerance test in WT mice fed low-fat (LF) or HF diet after 3 days treatment with vehicle, zaprinast, or 7ACC2 treatment. Values are presented as mean ± standard error of the mean. n = 7 to 10 per group. *D*, plasma insulin concentrations in WT mice fed LF or HF diet after 3 days treatment with vehicle, zaprinast, or 7ACC2 treatment. Values are presented as mean ± standard error of the mean. n = 8 to 18 per group. ∗*p* < 0.05 compared to LF vehicle. ∗∗*p* < 0.05 compared to LF and DIO vehicle. *E*, WT mice were fed a HF diet or LF control diet and then received 3 days of zaprinast or 7ACC2 treatment. An insulin bolus was injected 10 min prior to sacrifice. Liver insulin signaling was assessed using S473 phosphorylated–specific AKT antibodies by Western blot. The ratio of pAKT-s473/total AKT was quantified using densitometric analysis of band intensity, and the values are presented as mean ± standard error of the mean below the Western blot images. DIO, diet-induced obese.

To determine if these MPC inhibitors might elicit metabolic improvements similar to the effects of TZD-based MPC inhibitors, WT and LS-Mpc2−/− mice were fed a HF diet for 12 weeks and then treated with a single injection of 30 mg/kg zaprinast 18 h prior to assessing glucose tolerance. Zaprinast markedly improved glucose tolerance in DIO mice (Fig. 2*A*). Interestingly, zaprinast also further improved glucose tolerance in LS-Mpc2−/− mice, which were protected from the effects of DIO on glucose tolerance (Fig. 2*A*). This may indicate that zaprinast has peripheral effects that are not related to hepatic MPC inhibition, such as inhibiting the MPC in other tissues or stimulating muscle glucose uptake *via* PDE inhibition (26).

A single i.p. injection of 1 mg/kg 7ACC2 also markedly improved glucose tolerance in DIO mice in a glucose tolerance test (GTT) study performed 18 h later (Fig. 2*B*). In contrast to zaprinast, 7ACC2 did not further improve glucose tolerance in LS-Mpc2−/− mice. This suggests that both 7ACC2 and zaprinast enhance glucose tolerance in DIO mice; while the effects of 7ACC2 require MPC2 in hepatocytes, some of the effects of zaprinast may be mediated by effects independent of the MPC in hepatocytes or by other molecular mechanisms entirely.

The GTT, while useful, can be influenced by many variables and is not an indicator of insulin sensitivity *per se*. Treatment with zaprinast or 7ACC2 for 3 days improved insulin tolerance in an ITT performed in DIO WT mice (Fig. 2*C*). We also observed that at sacrifice, DIO mice treated with zaprinast or 7ACC2 had lower plasma insulin concentrations (Fig. 2*D*), which is consistent with a requirement for less insulin to maintain normoglycemia. The 3-day course of zaprinast or 7ACC2 treatment enhanced liver insulin signaling (insulin-induced phosphorylation of AKT-S473) following a bolus of insulin injected 10 min prior to sacrifice (Fig. 2*E*). Altogether, these results suggest that zaprinast and 7ACC2 may enhance insulin sensitivity in DIO mice.

### Metabolic improvements with acute MPC inhibition are not due to enhanced insulin sensitivity

**Figure 3:**
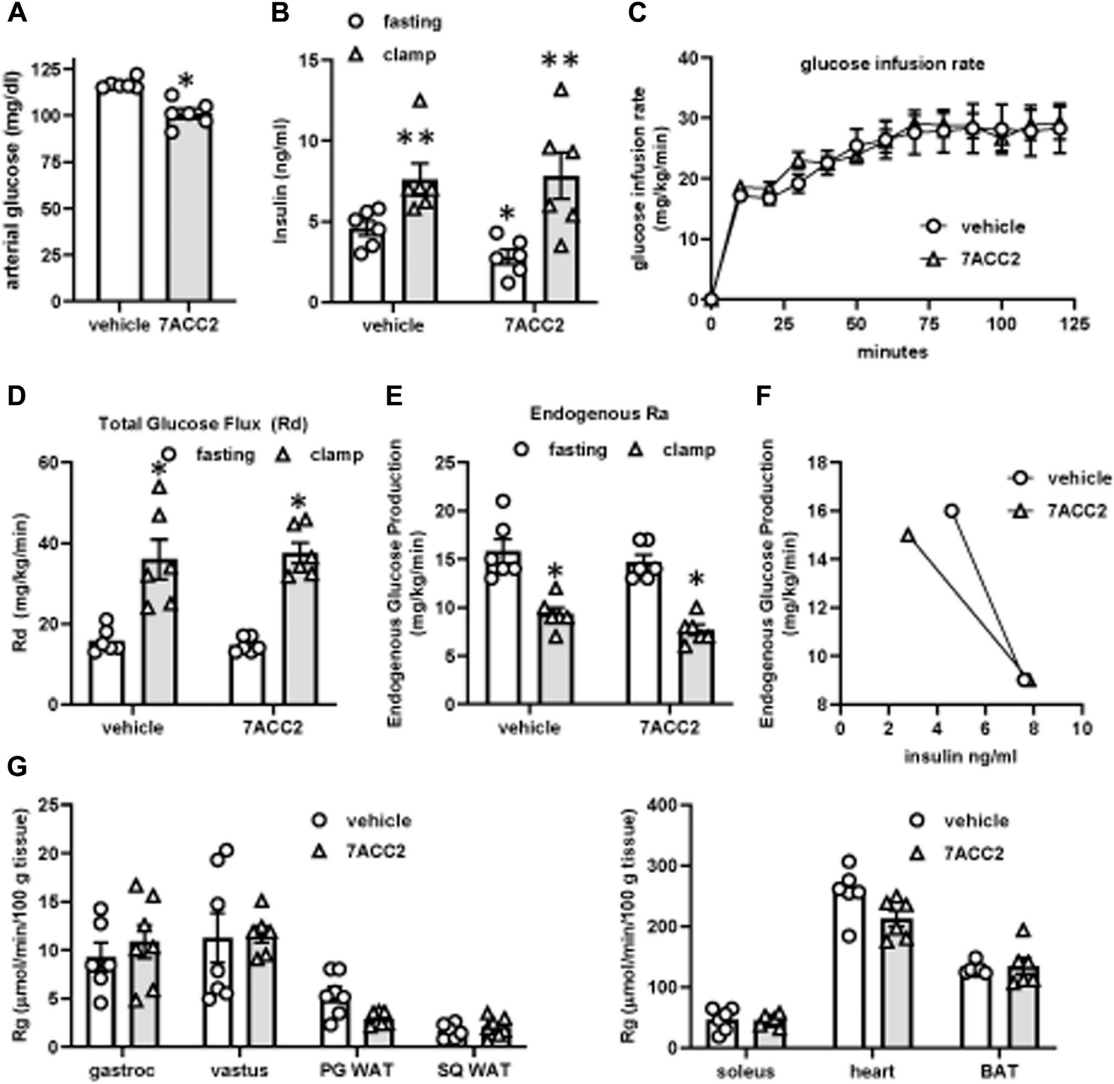
**7ACC2 does not improve insulin sensitivity in DIO mice.** C57BL/6J mice were fed a high-fat diet for 12 weeks and then treated with vehicle or 7ACC2 for 2 days before undergoing a hyperinsulinemic–euglycemic clamp. Graphs display: *A*, fasting arterial glucose; *B*, fasting or clamped insulin concentrations; *C*, glucose infusion rate; *D*, glucose flux (Rd); *E*, endogenous rate of appearance (Ra); *F*, Ra *versus* fasting and clamped insulin concentrations; and *G*, uptake of ^14^C 2-deoxyglucose into various insulin target tissues. Values are presented as mean ± standard error of the mean. n = (7) per group. ∗*p* < 0.05 compared to WT vehicle, ∗∗*p* < 0.01 *versus* fasting values in same treatment group. DIO, diet-induced obese.

To further examine whether 7ACC2 treatment resulted in improved insulin sensitivity, C57BL/6J DIO mice were treated with 7ACC2 for three consecutive days and then underwent hyperinsulinemic–euglycemic clamp. As observed previously, fasting blood glucose (Fig. 3*A*) and insulin (Fig. 3*B*) concentrations at the start of the clamp were reduced by treatment with 7ACC2. However, 7ACC2 did not affect glucose infusion rate (Fig. 3*C*) or total glucose flux (Fig. 3*D*). Endogenous glucose production (Ra) was also not statistically different in mice treated with 7ACC2 compared to vehicle controls (Fig. 3*E*). Because basal insulin concentrations were lower in mice treated with 7ACC2 compared to vehicle mice, glucose production rates as a function of plasma insulin were also calculated. Under fasting conditions, 7ACC2-treated mice had similar rates of Ra at a lower concentration of insulin. The slope of the response curve for mice treated with 7ACC2 was slightly shifted toward the left compared to the vehicle mice (Fig. 3*F*). Together, these findings are suggestive of metabolic improvements in response to 7ACC2 but do not definitively indicate improved insulin sensitivity. Finally, 7ACC2 treatment did not affect tissue 2-deoxyglucose uptake into muscle or fat (Fig. 3*G*). Thus, the acute effects of 7ACC2 on insulin sensitivity are very modest and likely suggest the metabolic improvement is due to improving glucose effectiveness or another mechanism of action.

### Acute MPC inhibition reduces glucose levels by suppressing hepatic glucose output

**Figure 4:**
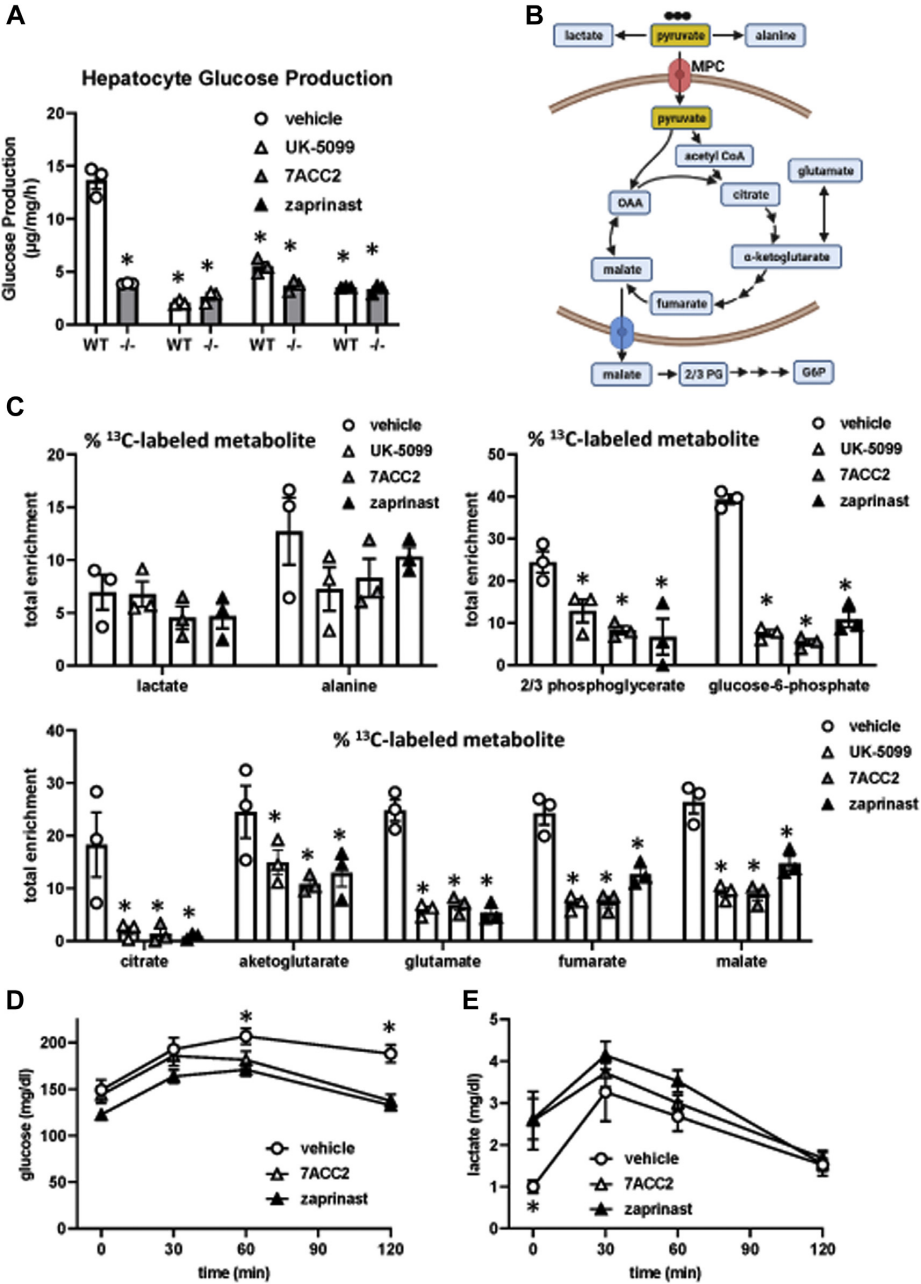
**7ACC2 and zaprinast suppress hepatocyte glucose production.***A*, pyruvate-stimulated hepatocyte glucose production assay in hepatocytes from littermate WT and LS-Mpc2−/− mice after treatment with vehicle, UK-5099, 7ACC2, or zaprinast. n = (3) per group. ∗*p* < 0.05 compared to pyruvate plus vehicle. *B*, ^13^C- and ^12^C-pyruvate was administered to WT hepatocytes treated with vehicle, UK-5099, 7ACC2, or zaprinast. Created with BioRender.com. *C*, total enrichment of ^13^C into the indicated metabolites measured by mass spectrometry after 3 h. Data presented as mean +standard error of the mean. n = (3) per group of a representative experiment (of two). ∗*p* ≤ 0.05 *versus* vehicle-treated hepatocytes. *D* and *E*, C57BL6/J mice were administered a single dose of vehicle, zaprinast, or 7ACC2, and a lactate/pyruvate tolerance test was conducted beginning 30 min later. Blood glucose (*D*) or lactate (*E*) concentrations are shown. ∗*p* < 0.05 compared to zaprinast and 7ACC2 concentrations at the same time point. MPC, mitochondrial pyruvate carrier.

We have previously shown that pharmacologic inhibition or genetic deletion of the MPC in hepatocytes attenuates pyruvate-stimulated glucose production (16), which could explain the *in vivo* metabolic effects of the MPC inhibitors. Thus, we tested the effect of these compounds on pyruvate-stimulated glucose production and found that zaprinast, 7ACC2, or the MPC inhibitor UK-5099 potently inhibited glucose output in isolated hepatocytes from WT mice (Fig. 4*A*). None of the MPC inhibitors had an effect on hepatic glucose output in hepatocytes isolated from LS-Mpc2−/− mice (Fig. 4*A*).

To confirm that the MPC inhibitors were affecting gluconeogenic flux using pyruvate as a substrate, we quantified incorporation of ^13^C-pyruvate into TCA cycle and gluconeogenic intermediates in isolated hepatocytes treated with MPC inhibitors (Fig. 4*B*). Treatment with 7ACC2, zaprinast, or UK-5099 did not affect incorporation of ^13^C-pyruvate into lactate or alanine (% ^13^C-labeled metabolite; Fig. 4*C*), which likely occurs in the cytosol (Fig. 4*B*). However, chemical inhibition of the MPC did increase the lactate pool size in the media (Fig. S2*A*). Incorporation into citrate and other TCA cycle intermediates was markedly disrupted by treatment with MPC inhibitors (Fig. 4*C*). Moreover, incorporation into the gluconeogenic intermediates, 2/3 phosphoglycerate and glucose-6-phosphate, was also markedly attenuated by MPC inhibition.

Consistent with an *in vivo* effect of the MPC inhibitors on gluconeogenesis, we found that administration of zaprinast or 7ACC2 to lean C57BL6/J mice 30 min prior to conducting a lactate/pyruvate tolerance test blunted the gluconeogenic response compared to vehicle-treated mice (Fig. 4*D*). Administration of zaprinast or 7ACC2 increased blood lactate concentrations in these mice prior to but not after administration of the lactate/pyruvate bolus (Fig. 4*E*). Administration of these compounds also had no impact on gene expression of gluconeogenic or lipogenic enzymes (Fig. S2*B*). When taken altogether, these data suggest that the metabolic improvement observed with acute, pharmacologic MPC inhibition *in vivo* is due to reduced hepatic glucose production using lactate/pyruvate as a substrate.

### RESPYR-based high-throughput screen for MPC modulators

**Figure 5:**
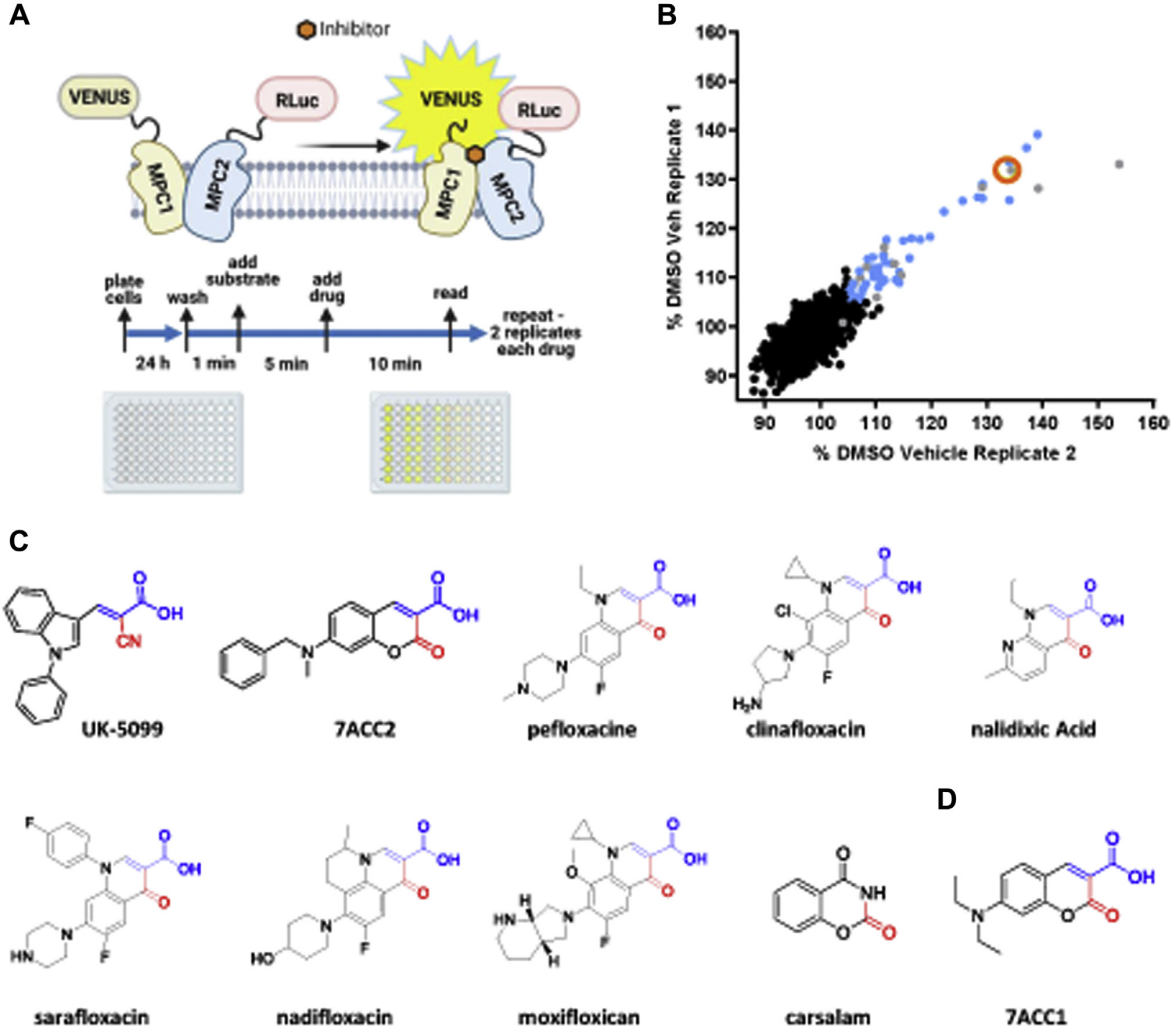
**A high-throughput screen identifies novel modulators of the MPC.***A*, the experimental workflow of the high-throughput screen of the Pharmakon 1600 library using the RESPYR system is shown. Created with BioRender.com. *B*, the first technical replicate is graphed on the y-axis, and the second replicate is graphed on the x-axis. Average UK-5099 reads for the screen are indicated with the *orange open circle*. Positive hits are indicated with *blue dots*. Compounds that altered the signal ratio in cells expressing only MPC2-RLuc8 in the absence of the acceptor, MPC1–Venus, are indicated in *gray* and were excluded from further analysis. *C* and *D*, the chemical structures of known MPC inhibitors and compounds in the Pharmakon 1600 library with similar chemical structures (*C*) or 7ACC1 (*D*) are shown. MPC, mitochondrial pyruvate carrier; RESPYR, reporter sensitive to pyruvate.

To identify novel MPC inhibitors, we screened the Pharmakon 1600 library of known drugs using the BRET-based MPC RESPYR system (Fig. 5*A*). Several compounds altered the BRET signal ratio in cells on a control plate that were not expressing the BRET donor protein (Venus-tagged MPC1), indicating false positivity (Fig. 5*B*). However, 35 compounds increased BRET activity only in cells expressing both the donor and acceptor proteins and were deemed to be positive hits (Fig. 5*B* and Table 1). Zaprinast, pioglitazone, and rosiglitazone are all included in the Pharmakon 1600 library and, as expected, were positive hits in our screen, which serves as validation for this approach.

**Table 1:** High-throughput screen hits

Drug name	Brand name	Known properties
Acrisorcin	Akrinol	Antifungal
Azilsartan kamedoxomil	Edarbi	Antihypertensive angiotensin II antagonist
Benzbromarone	Desuric	Uricosuric
beta-Naphthol	Microcidin	Anthelmintic, antiseptic
Candesartan cilexetil	Atacand	Angiotensin 1 receptor antagonist
Carprofen	Rimadyl	Antiinflammatory, analgesic
Carsalam	N/A	Analgesic
Clinafloxacin hydrochloride	N/A	Antibacterial
Dantrolene sodium	Dantrium	Muscle relaxant
Diclazuril	Clinacox	Coccidiostat
Dicumerol	Coumadin	Anticoagulant
Diflunisal	Dolobid	Analgesic, antiinflammatory
Esomeprazole potassium	Nexium	Gastric acid secretion inhibitor
Flufenamic acid	Arlef	Antiinflammatory, analgesic
Flumequine	Apurone	Antibacterial
Idebenone	N/A	Cognition enhancer, nootropic
Lornoxicam	Xefo	Analgesic, antiinflammatory
Mexeneone	Uvistat	Sunscreen
Monobenzone	Benoquin	De-pigmentor
Moxifloxcian hydrochloride	Avelox	Antibacterial
Nadifloxacin	Acuatim	Antibacterial
Nalidixic acid	Neggram	Antibacterial
Nitrofurantoin	Furadantin	Antibacterial
Oxaprozin	Daypro	Antiinflammatory
Pefloxacin mesylate	N/A	Antibacterial, antiproliferative
Phenyl aminosalicylate	Phenypastebamin	Antibacterial (tuberculostatic)
Pioglitazone hydrochloride	Actos	Antidiabetic
Pregnenolone succinate	formula 405	Glucocorticoid, antiinflammatory
Racecadotril	Tiorfan	Antidiarrheal
Rosiglitazone maleate	Avandia	Antidiabetic
Sarafloxacin hydrochloride	Saraflox	Antibacterial
Sulfadimethoxine	Madribon	Antibacterial
Telmisartan	Micardis	Antihypertensive, angiotensin II blocker
Torsemide	Demadex	Diuretic, inhibits Na/K/2Cl carrier system
Zaprinast	N/A	cGMP phosphodiesterase inhibitor

Several compounds deemed positive hits were validated using kinetic RESPYR assays (Figs. S3 and S4). However, some of the compounds displayed responses of considerably smaller magnitude compared to positive controls (Fig. S3) or that required micromolar concentrations (Fig. S4).

To aid in prioritizing which hits to further pursue, we built a pharmacophore model that represents the geometrical and chemical features of 7ACC2 (Fig. 5*C*). To prioritize compounds from the hits identified in the high-throughput screen for further testing, we screened the 35 hits against the pharmacophore model. The top scored compounds were the six quinolone antibiotics shown in Figure 5*C*. These compounds share a common pharmacophore with 7ACC2 because they possess a Michael acceptor unit (highlighted in *blue*, Fig. 5*C*). In addition, the screened compound carsalam, a nonsteroidal anti-inflammatory agent, also possesses a similar chemical structure (Fig. 5*C*). Finally, 7ACC1, which is sold as a plasma membrane MCT inhibitor, shares the same coumarin pharmacophore with 7ACC2 (Fig. 5*D*) but was not present in the chemical library. Based on this common pharmacophore, the quinolone compounds, carsalam, and 7ACC1 were selected for further validation.

**Figure 6:**
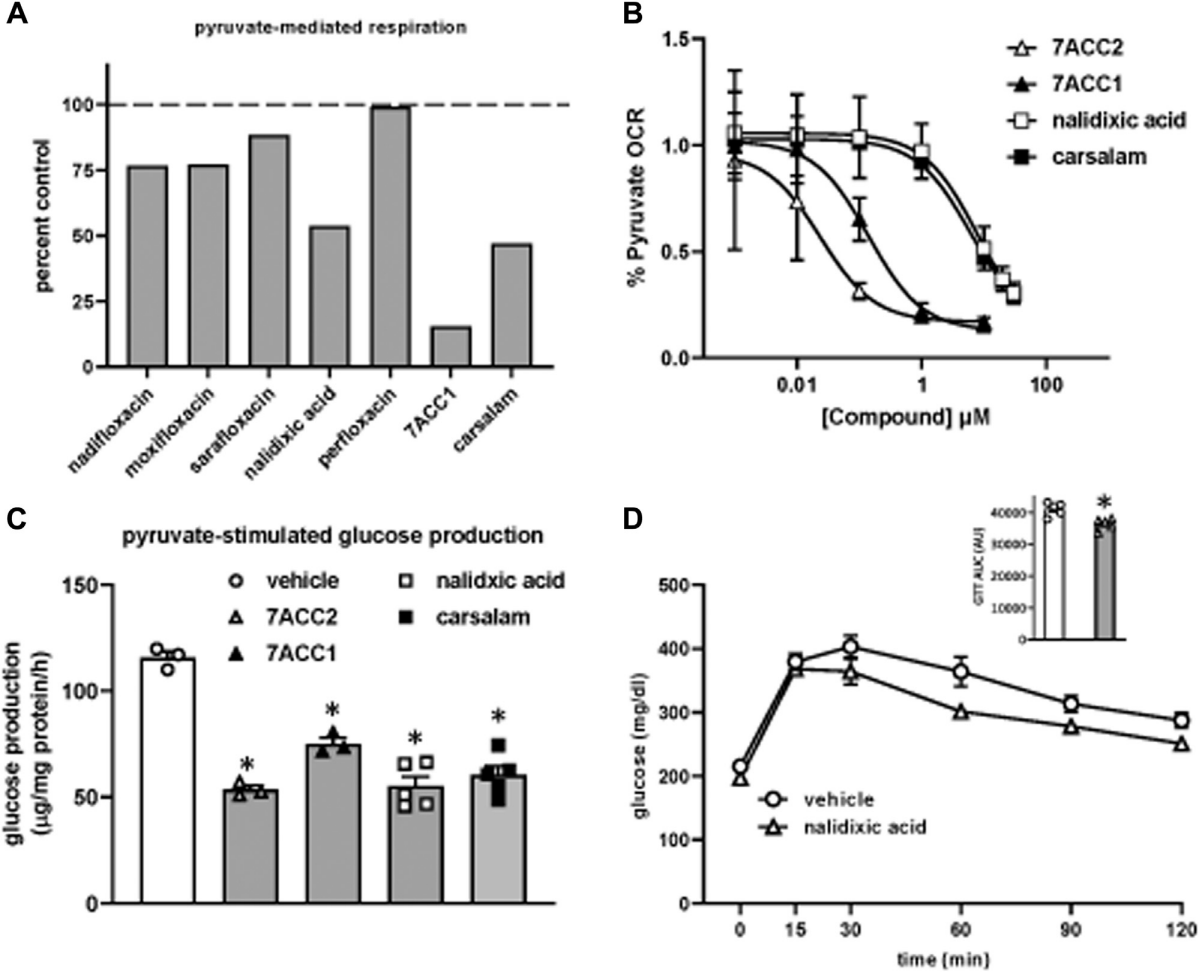
**The novel MPC inhibitors nalidixic acid, 7ACC1, and carsalam inhibit pyruvate-stimulated mitochondrial respiration, attenuate hepatocyte glucose production, and improve glucose tolerance in DIO mice.***A*, pyruvate-stimulated mitochondrial respiration with 10 μM of the indicated compounds are shown. Respiration values were normalized to vehicle control treatment. *B*, mitochondrial respiration dose–response curves of 7ACC2, 7ACC1, nalidixic acid, and carsalam. *C*, the effects of 7ACC2, 7ACC1, nalidixic acid, and carsalam on pyruvate-stimulated hepatocyte glucose production are shown. *D*, C57BL/6J mice were fed a high-fat diet for 14 weeks and then administered a daily dose of nalidixic acid or vehicle control for 3 days prior to glucose tolerance testing. Area under the curve was calculated and is displayed inset in the bar graph. ∗*p* < 0.05 compared to vehicle. DIO, diet-induced obese; OCR, oxygen consumption rate.

We tested the ability of some of the quinolones, carsalam, and 7ACC1 to inhibit mitochondrial respiration using pyruvate as a metabolic substrate. We found that nalidixic acid, 7ACC1, and carsalam were the most effective of these compounds at 10 μM (Fig. 6*A*). Respiration studies comparing increasing doses of these top compounds revealed that 7ACC1 was nearly as potent as 7ACC2 (Fig. 6*B*) with an IC50 similar to UK-5099 (data not shown). Nalidixic acid and carsalam were relatively less potent but inhibited pyruvate-mediated respiration by ∼50% at 10 μM (Fig. 6*B*). We then assessed the ability of these novel MPC inhibitors to suppress hepatocyte glucose production. We found that 7ACC2, 7ACC1, nalidixic acid, and carsalam potently inhibited glucose production by isolated hepatocytes at 10 μM concentrations (Fig. 6*C*).

Finally, we treated DIO mice with the nalidixic acid sodium salt, which is water soluble, for 3 days and then conducted GTT analyses. Nalidixic acid tended to reduce fasting glucose levels compared to vehicle controls, but this did not reach statistical significance (Fig. 6*D*). However, similar to the other MPC inhibitors, glucose tolerance following a glucose bolus was significantly improved by nalidixic acid treatment (Fig. 6*D*).

## Discussion

Herein, we sought to determine the potential of using MPC inhibitors for the pharmacologic treatment of metabolic derangements in a mouse model. We confirm that zaprinast and 7ACC2 are direct and *bona fide* MPC inhibitors and demonstrate that these compounds acutely lower plasma glucose and insulin concentrations and improve glucose tolerance in DIO mice. The metabolic improvements observed with acute MPC inhibition are likely explained by the observed effects on pyruvate-driven gluconeogenesis, which is known to be overactive in diabetic liver, rather than an overt effect on insulin sensitivity that has been observed in previous studies with a TZD-based MPC inhibitor (7, 11). This could be due to the acute duration of MPC inhibition in this study or due to other effects of the TZD-based drugs. Our results demonstrate the potential for therapeutics that interact with the MPC and modulate its activity for treating a variety of obesity-related metabolic diseases.

Metabolic improvements seen with MPC inhibitors are potentially due to a variety of mechanisms that remain to be fully delineated. Studies conducted herein, and previous work with liver MPC–null mice, suggest that attenuation of hepatic glucose production contributes to the glucose-lowering effect (16, 17). Pyruvate entry into the committed steps of the gluconeogenic and *de novo* lipogenic pathways requires mitochondrial metabolism of this substrate (Fig. 4*B*). Alternatively or in addition, inhibition of pyruvate entry into mitochondria may also enhance the use of other mitochondrial substrates. Indeed, MPC-deficient hepatocytes exhibited enhanced reliance on alanine and glutamine utilization for gluconeogenesis and increased palmitate oxidation *in vitro* (16, 17). Herein, we show that the acute effects of 7ACC2 on glucose tolerance require intact MPC in hepatocytes because liver MPC2−/− mice do not show further improvement in glucose tolerance after 7ACC2 treatment. Moreover, the metabolic improvements that are shown do not correspond to a dramatic improvement in insulin sensitivity in hyperinsulinemic clamp studies or reduced expression of gluconeogenic enzymes (Fig. S2). Rather, it is likely that much of the effect of acute MPC inhibition is driven by metabolic suppression of hepatic glucose production.

Owing to the poor solubility and limited half-life of zaprinast and 7ACC2, only acute studies were conducted to examine metabolic effects. Possibly with longer term treatment, MPC inhibition in tissues other than the liver could lead to insulin sensitization and contribute to the beneficial effects. Although somewhat counterintuitive, others have shown that MPC inhibition with TZDs or UK-5099 increased glucose uptake in skeletal muscle myocytes *in vitro*, potentially *via* increased AMP activated kinase phosphorylation in skeletal muscle (4). MPC inhibition or knockout may also enhance brown adipocyte differentiation and beiging of white adipose tissue depots (3, 20). However, inhibition of the MPC in pancreatic beta cells may directly suppress glucose-stimulated insulin secretion (14, 27, 28), which would potentially counteract glucose-lowering effects on gluconeogenesis. Additionally, cardiac-specific knockout of the MPC results in cardiomyopathy in mice (25, 29, 30), suggesting that strong inhibition in the myocardium should be avoided. Further work is needed to fully delineate the mechanistic aspects of the observed improvements with MPC inhibitor administration and define whether liver-specific MPC inhibition might be preferable.

Both zaprinast and 7ACC2 have been associated with effects on other transporters and metabolic enzymes. Zaprinast was identified as an inhibitor of PDE enzymes many decades ago (31). Previous work has demonstrated that it also acts as an agonist for GPR35 (32) and as an inhibitor of glutaminase (33). Zaprinast has several reactive groups and may be promiscuously interacting with a variety of proteins. There is evidence that PDE inhibition may stimulate glucose uptake by skeletal muscle (26), and our studies with the LS-Mpc2−/− mice indicate that zaprinast has effects on glucose tolerance that are independent of liver MPC2 (Fig. 2*A*). In previous work, when zaprinast was acutely administered to rats, muscle glucose uptake was increased in clamp studies (26). But, the authors also observed a significant rise in plasma lactate concentrations that likely came from organs other than skeletal muscle, which is consistent with the inhibitory effects of zaprinast on the MPC.

On the other hand, the effects of 7ACC2 on glucose tolerance seem to require MPC2 in liver (Fig. 2*B*). 7ACC2 was originally described as an inhibitor of cellular lactate import, but not efflux, believed to be mediated by inhibiting the plasma membrane monocarboxylate transporter (34). However, recent work suggested that this purported selective inhibition was actually explained by accumulation of lactate secondary to MPC inhibition (23). Herein, the use of the RESPYR assay demonstrates that 7ACC2 directly interacts with the MPC and seems as potent as the prototypical MPC inhibitor UK-5099. The data showing that 7ACC2 improves glucose tolerance in a manner that requires liver MPC2 are also indicative of this selectivity. While zaprinast and 7ACC2 may be useful as tool compounds, their potential for off-target effects (zaprinast), poor solubility, and short half-life limited the duration of studies presented herein and make them poor candidates to advance in development as therapeutics.

The current screen provides multiple hits that can be used as a starting point toward discovering more potent and efficacious MPC inhibitors. Although the newly identified drugs are weak MPC inhibitors, they are amenable to chemical optimization to improve their pharmacodynamics toward MPC and benefit from their good bioavailability, low toxicity, and favorable pharmacokinetics. One of the primary targets of quinolones in bacteria is the topoisomerase IV–DNA cleavage complex. The keto acid group of quinolones facilitates binding to topoisomerase through a water–Mg^2+^ ion bridge (35). Interestingly, quinolones have been associated with hypoglycemia especially when used with other antidiabetic agents (36, 37). Additionally, the pharmacophore modeling also suggested that carsalam and 7ACC1 interact with the MPC by a similar mechanism, and inhibition was validated experimentally. Chemical optimization of these compounds can be used to enhance the potency and selectivity of compounds toward MPC. Novel small-molecule inhibitors of the MPC will be needed for potential utility in treating not only diabetes but also other diseases including nonalcoholic steatohepatitis, cancer (23), neurological diseases (38), and even hair loss (39). Identification of new MPC inhibitors could benefit drug development programs for these diseases and other conditions associated with altered metabolism.

In summary, our results demonstrated that chemically diverse MPC inhibitors improve dysregulated metabolism in DIO mice, likely by suppressing hepatic glucose production. Furthermore, by using the results of a screen of a chemical library, future chemical optimization of these drugs combined with ligand-based design approaches will pave the way to identification of novel MPC inhibitors with excellent pharmacokinetics and potential for liver-specific effects.

## Experimental procedures

### High-throughput screen

A high-throughput screen of the Pharmakon 1600 library was designed using a previously reported BRET-based MPC activity assay (RESPYR) (Fig. 1*A*) (24). HEK-293 cells constitutively expressing MPC1–Venus and MPC2-RLuc8 fusion proteins, or the MPC2-RLuc8 construct only as a negative control, were plated 24 h prior to assays in clear-bottom, white 96-well plates and maintained at 37 °C, 5% CO_2_ (Fig. 1*A*). Liquid media were aspirated using an ELx405 Plate Washer. A white sticker was placed on the bottom of each plate immediately prior to each assay. Using a BiomekFX liquid handler, BRET substrate (coelenterazine h) was added at 2× concentration (5 μM final concentration) in PBS supplemented with CaCl and MgCl_2_ to the cells, 5 min after which each well received one of the Pharmakon 1600 library compounds (MicroSource Discovery Systems; provided by the Washington University High-Throughput Screening Center) at 2× concentration in PBS supplemented with CaCl and MgCl_2_ (10 μM final concentration). Cells were then incubated for 9 min. Following a 1-min dark adaptation, bioluminescence measurements were taken using 485 nm (Donor; Rluc8) and 535 nm (Acceptor; Venus) filters with a PerkinElmer Envision plate reader. Positive (UK-5099) and negative (0.5% dimethyl sulfoxide [DMSO]) controls were included on each plate. Data were normalized to vehicle (DMSO) controls on a per plate basis and expressed as a percent of controls.

Two technical replicates were performed for each compound. In addition, all compounds were also tested on a single control plate with cells expressing only the BRET donor construct, hMPC2-RLuc8, in the absence of the BRET acceptor, hMPC1–Venus, to detect nonspecific changes in donor signal upon compound addition. Compounds were called as positives if both replicates showed a greater than 5% increase in BRET signal. Compounds that altered the hMPC2-RLuc8 signal in the absence of the hMPC1–Venus acceptor are labeled in gray (Fig. 1*B*) and were considered negative. Postscreen verification of positive hits was carried out using kinetic RESPYR assays with dose–response curves, as described (9).

### MPC inhibitors

Zaprinast, UK-5099, moxifloxacin, and nalidixic acid (sodium salt) were obtained from Millipore Sigma. 7ACC2 was obtained from Cayman Chemical. 7ACC1, nalidixic acid, nadifloxacin, sarafloxacin, and carsalam were obtained from MedChem Express. Pefloxacin was purchased from CP Lab Safety.

### Gal4-PPARγ luciferase reporter assays

The luciferase cotransfection assays were performed in a 4-day format. HEK293T cells (ATCC; CRL-1573) were seeded in Corning 3598 96-well plates at a density of 20,000 cells per well in 50 μl of Dulbecco's modified Eagle's medium (DMEM) (Gibco) supplemented with 5 mM L-glutamine (Corning) and 10% fetal bovine serum (FBS) (Gemini Bio) and allowed to settle overnight in a 5% CO_2_ incubator at 37 °C. On day 2, transfection of the cells was performed by incubating Opti-MEM (Gibco), lipofectamine2000 (ThermoFisher Scientific), 100 ng/μl pG-luc (Promega; E2440), and 50 ng/μl chimeric Gal4-DBD fused to nuclear receptor–LBD in pBIND[Zeo] for 30 min. Twenty-five microliters of the transfection mixture was then added to the corresponding well, and the cells were gently centrifuged and placed back in the incubator overnight. The following day, cells were treated with compound or DMSO control by adding 4× treatment in DMEM media with 0.4% DMSO in a volume of 25 μl so that the final volume in each well was 100 μl. Cells were briefly centrifuged and incubated overnight. On the final day, 75 μl of OneGlo Luciferase Reagent (Promega) was added to each well and pipetted vigorously to lyse the cells. One-hundred microliters of each sample was then transferred to a Corning 3912 opaque white 96-well plate, and luminescence was read on a Biotek Neo Alpha Instrument. Data were analyzed using GraphPad Prism. Luminescence values were normalized to DMSO (ratio relative luciferase units drug:DMSO), and then compound concentrations were log-transformed and curves were fitted by nonlinear regression (agonist mode). Data are represented by mean (n = 4–8) ± SEM.

### Animals

All experiments performed in mice were approved by the Institutional Animal Care and Use Committee at the Washington University School of Medicine. Liver-specific *Mpc2* knockout (LS-*Mpc2*−/−) or cardiac-specific *Mpc2* knockout (CS-*Mpc2*−/−) mice were generated as described using LoxP technology with Cre expression driven by the albumin gene promoter (16) or myosin light chain 2v promoter (25), respectively. Control mice were littermates not expressing Cre recombinase (fl/fl).

For HF diet studies, LS-Mpc2−/− and littermate control mice were switched from standard chow to a 60% HF diet (Research Diets Inc, #D12492) at 7 to 8 weeks old, and experiments were performed after 12 weeks on diet. For studies not in the *Mpc2* null background, C57BL/6J DIO mice were purchased from Jackson Laboratory (cat #380050) after 9 weeks of feeding a 60% fat diet (Research Diets Inc, #D12492) and were maintained on the same diet for the indicated times. When included in the experimental design, low-fat (LF) diet C57BL/6J control mice were also purchased from Jackson Laboratory (cat #380056) and fed 10% LF control diet (Research Diets Inc, #D12450B). LF diet controls were matched in age to DIO comparators.

### Drug treatments

Zaprinast or 7ACC2 was solubilized in 25% DMSO/75% saline and injected i.p. at a dose of 30 mg/kg or 10 mg/kg, respectively, 18 h prior to GTT and ∼22 h prior to sacrifice. Nalidixic acid (sodium salt) was dissolved in 0.9% saline and injected i.p. at a dose of 30 mg/kg once daily for 3 days and then again 4 h prior to the GTT. Control mice received a similar volume of vehicle solution i.p.

### Glucose, insulin, and lactate/pyruvate tolerance tests

Mice were fasted for 4 h, and glucose, insulin, or lactate/pyruvate tolerance tests were performed as described (16). Briefly, mice were injected i.p. with 1 g/kg glucose (GTT), 0.75 U/kg humulin (ITT), or lactate (900 mg/kg) and pyruvate (100 mg/kg) (lactate/pyruvate tolerance test) in saline. Blood glucose levels were measured from a drop of tail blood using a OneTouch glucometer at 0, 15, 30, 60, 90, and 120 min.

### Hyperinsulinemic–euglycemic clamp studies

Hyperinsulinemia clamp studies were performed by the Vanderbilt Mouse Metabolic Phenotyping Center. Male C57BL/6J DIO mice were purchased from Jackson Laboratory (cat #380050) after 9 weeks of feeding a 60% HF diet (Research Diets Inc, #D12492) and were maintained on this diet for the duration of the study. After 11 weeks on the diet, the right jugular vein and left carotid artery were surgically catheterized, and mice recovered for 1 week. Mice were randomized by weight to vehicle (25% DMSO in saline; n = 7) or drug (7ACC2, 5 mg/kg i.p.; n = 7) and were treated for three consecutive days (day 0, day 1, and day 2). On day 2, a hyperinsulinemic–euglycemic clamp was performed on 5-h fasted conscious mice using the protocol established at the Vanderbilt Mouse Metabolic Phenotyping Center (40, 41). A continuous infusion of 2.5 mU/min/kg body weight insulin was carried out, and this dose was chosen to probe a predicted improvement in liver insulin action. At t = −90 min, a primed-continuous infusion of HPLC-purified 3-[^3^H]-glucose (PerkinElmer) was begun and maintained throughout the experiment. Euglycemia (∼9 mmol/l) was maintained by measuring blood glucose every 10 min starting at t = 0 min and infusing 50% dextrose as necessary. Additional blood was taken at t = 80, 90, 100, 110, and 120 min and processed to determine plasma 3-[^3^H]-glucose. A 12-μCi bolus of 2-[^14^C]-deoxyglucose (2-[^14^C]DG) was given at t = 120 min. Blood samples were obtained at t = 122, 135, 145, and 155 min and processed to determine plasma 2-[^14^C]DG. Mice received saline-washed erythrocytes from donors beginning at t = 0 min and continuously throughout the clamp to prevent a fall of >5% hematocrit. Rates of glucose infusion, hepatic glucose output, and tissue 2-DG uptake were calculated. At 155 min, pentobarbital anesthesia was administered to anesthetize the mice, and tissue was collected and flash frozen.

### Hepatocyte studies

Primary hepatocytes were isolated from fl/fl (WT) or LS-*Mpc2−/−* mice by cannulation of the portal vein, and collagenase digestion was performed as described (16). Cells were then counted and plated on collagen-coated 12-well plates in DMEM medium containing 10% FBS, 1× Pen–Strep, and 1× amphotericin B.

Hepatocyte glucose production assays were performed as described (16). The morning after isolation, cells were washed 2× with PBS and starved for 2 h in Hank's Buffered Saline Solution (HBSS) (containing 127 mM NaCl, 3.5 mM KCl, 0.44 mM KH_2_PO_4_, 4.2 mM NaHCO_3_, 0.33 mM Na_2_HPO_4_, 1 mM CaCl_2_, and 20 mM Hepes, pH 7.4). HBSS was removed, and cells were washed in fresh HBSS and then treated for 3 h in HBSS containing glucagon (100 ng/ml) alone or with 5 mM sodium pyruvate in the absence or presence of either 2.5 μM UK-5099 or 10 μM zaprinast, 7ACC2, or other candidate inhibitors. After the 3-h incubation, media were collected, and glucose concentrations were measured using a glucose oxidase–based glucose assay kit (Sigma Aldrich). Glucose concentrations were normalized to cell protein amount, which was measured by Micro BCA kit (ThermoFisher Scientific).

For studies using ^13^C-labeled pyruvate, the morning after isolation, cells were rinsed with PBS twice. Cells were starved for 2 h in HBSS (containing 127 mM NaCl, 3.5 mM KCl, 0.44 mM KH2PO4, 4.2 mM NaHCO3, 0.33 mM Na2HPO4, 1 mM CaCl2, and 20 mM Hepes, pH 7.4). HBSS was removed, and cells were washed with fresh HBSS and then treated for 3 h in HBSS containing glucagon (100 ng/ml) and 5 mM pyruvate (unlabeled) or mixed 2.5 mM pyruvate with 2.5 mM ^13^C-labeled pyruvate and in the absence or presence of either 2.5 μM UK-5099 or 10 μM zaprinast or 10 μM 7ACC2. In parallel, a separate group of cells were treated with 10% FBS-DMEM solution containing glucagon (100 ng/ml) alone (for background assessment) or 5 mM ^13^C-labeled pyruvate in 600 μl per well and allowed to incubate for 3 h. Studies were conducted in triplicate in a 6-well plate. Media (100 μl) were collected before harvesting cells. Samples were centrifuged at 14,000*g* and 4 °C for 10 min, and the supernatant was stored at −80 °C until metabolite analysis. For cell harvest and extraction, cells were washed twice with PBS and twice with HPLC-grade water. Cold HPLC-grade methanol was used for quenching, and cells were scraped and the lysates transferred to sterile Eppendorf tubes. Samples were dried in a SpeedVac for 2 to 6 h. The dried samples were reconstituted in 1 ml of cold methanol:acetonitrile:water at a 2:2:1 ratio and subjected to three cycles of vortexing, freezing in liquid nitrogen, and 10 min of sonication at 25 °C. Samples were then stored at −20 °C for 1 h. After this, samples were centrifuged at 14,000*g* and 4 °C. The protein content of pellets was measured by Micro BCA kit (ThermoFisher Scientific). Supernatants were transferred to new tubes and dried by SpeedVac for 2 to 5 h. After drying, we added 1 μl of water:acetonitrile (at a ratio of 1:2) per 2.5 μg of cell protein in pellets obtained after extraction. Samples were subjected to two cycles of vortexing and 10 min of sonication at 25 °C. Next, we centrifuged at 14,000*g* and 4 °C for 10 min, transferred supernatant to LC vials, and stored at −80 °C until MS analysis.

### Metabolite analysis by LC/MS

Ultrahigh-performance LC/MS was performed with a Thermo Scientific Vanquish Horizon UHPLC system interfaced with a Thermo Scientific Orbitrap ID-X Tribrid Mass Spectrometer. Hydrophilic interaction liquid chromatography separation was accomplished by using a HILICON iHILIC-(P) Classic column (HILICON AB) with the following specifications: 100 mm × 2.1 mm, 5 μm. Mobile-phase solvents were composed of A = 20 mM ammonium bicarbonate, 0.1% ammonium hydroxide (adjusted to pH 9.2), and 2.5 M medronic acid in water:acetonitrile (95:5) and B = 2.5 M medronic acid in 95:5 acetonitrile:water. The column compartment was maintained at 45 °C for all experiments. The following linear gradient was applied at a flow rate of 250 l min^−1^: 0 to 1 min: 90% B, 1 to 12 min: 90 to 35% B, 12 to 12.5 min: 35 to 25% B, 12.5 to 14.5 min: 25% B. The column was re-equilibrated with 20 column volumes of 90% B. The injection volume was 2 l for all experiments.

Data were collected with the following settings: spray voltage, −3.5 kV; sheath gas, 35; auxiliary gas, 10; sweep gas, 1; ion transfer tube temperature, 275 °C; vaporizer temperature, 300 °C; mass range, 67 to 1500 Da, resolution, 120,000 (MS1), 30,000 (MS/MS); maximum injection time, 100 ms; isolation window, 1.6 Da. LC/MS data were processed and analyzed with the open-source Skyline software (42). Natural abundance correction of ^13^C for tracer experiments was performed with AccuCor (43).

### Mitochondrial respiration

Hearts were removed from WT mice after CO_2_ asphyxiation and homogenized in buffer containing 250 mM sucrose, 10 mM Tris base, and 1 mM EDTA (pH = 7.4) by 8 to 10 passes of a glass-on-glass Dounce homogenizer on ice. Homogenates were centrifuged at 1000*g* for 5 min at 4 °C to pellet nuclei and undisrupted cells. The supernatants were then centrifuged at 10,000*g* for 10 min at 4 °C to enrich for mitochondria, and this mitochondrial pellet was washed and repelleted twice in fresh sucrose/Tris buffer. The mitochondrial pellet was then solubilized in ∼150 l of Mir05 respiration buffer (0.5 mM EGTA, 3 mM MgCl, 60 mM lactobionic acid, 20 mM taurine, 10 mM KH2PO4, 20 mM Hepes, 110 mM sucrose and 1 g/l of fatty acid free bovine serum albumin [BSA]; pH 7.1). Mitochondrial protein content was then measured by BCA, and 50 μg of mitochondrial protein was added to an Oxygraph O2K chamber (Oroboros Instruments), with a total volume of 2 ml Mir05 buffer. Respiration was stimulated with 5 mM pyruvate/2 mM malate and 2 mM ADP. After obtaining steady-state respiration measurements, compounds were added to the chamber at the indicated concentrations. Succinate (5 mM) was then added to determine inhibitor specificity toward pyruvate-stimulated respiration. Steady-state rates of oxygen consumption were assessed for 1 to 2 min before addition of subsequent substrate or inhibitor. Oxygen consumption rates were calculated from the change in oxygen concentration over time and normalized to 50 μg of mitochondria within the chamber. For the experiments shown, respiratory control ratios were between 7 and 10, indicating high-quality mitochondrial preparations.

### Western blotting for insulin signaling

Mice were injected i.p. with 0.9% NaCl or 5 mU/g insulin in 0.9% NaCl and sacrificed 10 min after injection. Tissues were harvested and snap frozen in liquid nitrogen and stored at −80 °C until analyzed. Liver tissue ∼50 mg was homogenized using a bead homogenizer lysis buffer consisting of 15 mM NaCl, 25 mM Tris base, 1 mM EDTA, 0.2% NP-40, and 10% glycerol supplemented with 1× complete protease inhibitor cocktail and phosphatase inhibitors (1 mM Na_3_VO_4_, 1 mM NaF, and 1 mM PMSF). Protein concentrations were measured by BCA assay, and 50 g of protein was electrophoresed on 4% to 15% polyacrylamide gels and transferred onto 0.45 m poly(vinylidene fluoride) membranes. Membranes were then blocked in 5% BSA in Tris-buffered saline with Tween-20 (TBST) for 1 h. Primary antibodies were then used at 1:1000 in 5% BSA-TBST overnight while rocking at 4 °C. Antibodies for phosphorylated AKT S473 and total AKT were from Cell Signaling (4060 and 4691, respectively), while the antibody for -Tubulin was from Sigma (T5168). Primary antibodies were incubated overnight at 4 °C. After primary antibody incubation, membranes were washed with TBST and probed with near-IRDye secondary antibodies (926-32213 and 926-68072) in 5% BSA-TBST for 1 h, washed, and then developed on a LiCor Odyssey imager. AKT activation by insulin was quantified by measuring the densitometry of pAKT-S473 and total AKT using LiCor ImageStudio Lite software.

### Liver RNA expression

Liver tissue was collected at sacrifice and snap frozen in liquid nitrogen. Total RNA from livers or hepatocytes was isolated using RNA-Bee (Tel-Test). Complementary DNA was synthesized by using a reverse transcription kit (Invitrogen), and real-time PCR was performed using an ABI PRISM 7500 sequence detection system (Applied Biosystems) and a SYBR green master mix. Arbitrary units of target mRNA were normalized by the comparative Ct method to levels of 36B4 mRNA.

### Statistical analyses

All data are presented as mean ± standard error of the mean, with statistical significance defined as *p* < 0.05. RESPYR data were analyzed by repeated measures ANOVA in GraphPad Prism. Other data were analyzed by one-way or two-way ANOVA as appropriate. *Post hoc* analysis was performed using Tukey’s multiple comparison tests.

## Data availability

All data generated during these studies are included in the text, figures, and tables of this article and electronic supplementary material. Source data or materials will be supplied by the corresponding author with reasonable request.

## Supporting information

This article contains supporting information.

## Conflict of interest

Brian Finck is a shareholder and member of the scientific advisory board of Cirius Therapeutics, which is developing the MPC inhibitor MSDC-0602K for clinical use. The other authors declare that they have no conflicts of interest with the contents of this article.
