# Supplementary material for: Mitochondrial pyruvate carrier inhibitors improve metabolic parameters in diet-induced obese mice
Source: J Biol Chem. 2021 Dec 30;298(2):101554. doi: 10.1016/j.jbc.2021.101554 (PMC8808181; doi:10.1016/j.jbc.2021.101554)
Supplement: Supplemental Figures S1–S4 [file mmc1.pptx]

## Slide 1
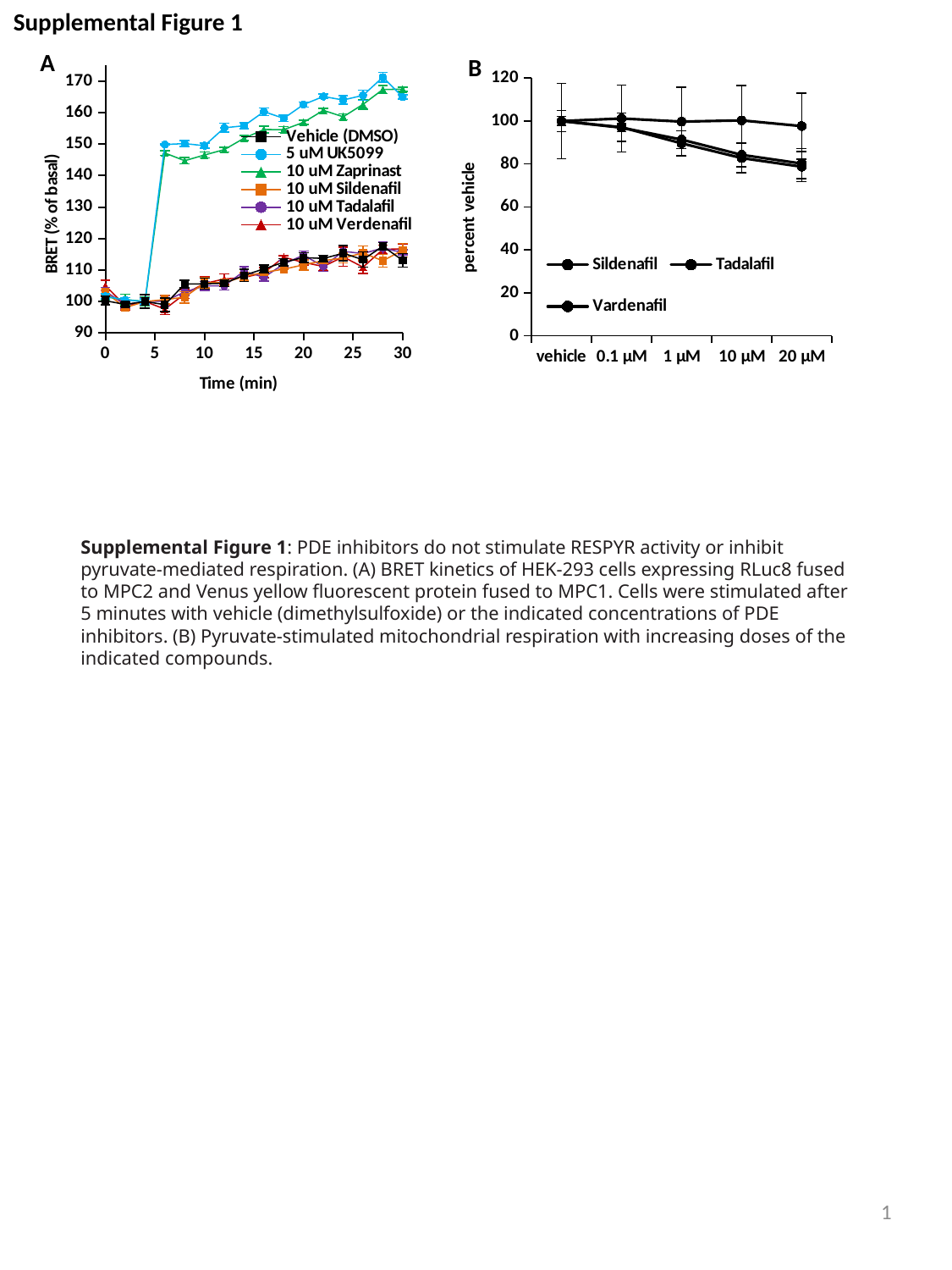

Supplemental Figure 1
A
B
### Chart
| Category | Vehicle (DMSO) | 5 uM UK5099 | 10 uM Zaprinast | 10 uM Sildenafil | 10 uM Tadalafil | 10 uM Verdenafil |
|---|---|---|---|---|---|---|
### Chart
| Category | Sildenafil | Tadalafil | Vardenafil |
|---|---|---|---|
| vehicle | 100.0 | 100.0 | 100.0 |
| 0.1 µM | 96.92269842010256 | 97.0998419936036 | 101.17406483617508 |
| 1 µM | 91.36241070540322 | 89.61981377114918 | 99.74469545459081 |
| 10 µM | 84.25821670691535 | 82.76720338658046 | 100.29328371364967 |
| 20 µM | 80.21707571986025 | 78.74850608181345 | 97.64012623491192 |Supplemental Figure 1: PDE inhibitors do not stimulate RESPYR activity or inhibit pyruvate-mediated respiration. (A) BRET kinetics of HEK-293 cells expressing RLuc8 fused to MPC2 and Venus yellow fluorescent protein fused to MPC1. Cells were stimulated after 5 minutes with vehicle (dimethylsulfoxide) or the indicated concentrations of PDE inhibitors. (B) Pyruvate-stimulated mitochondrial respiration with increasing doses of the indicated compounds.
1

## Slide 2
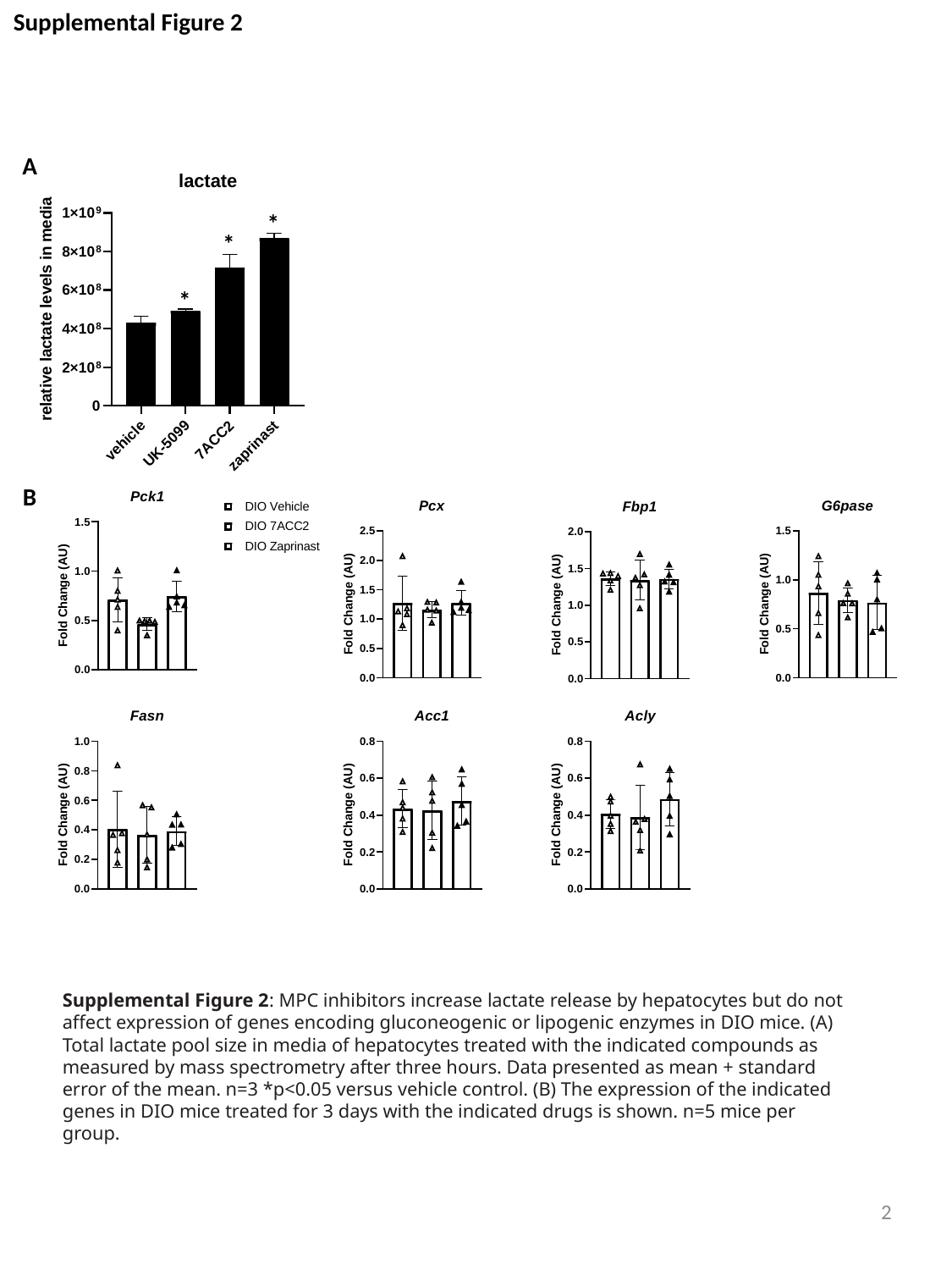

Supplemental Figure 2
A
*
*
*
B
Supplemental Figure 2: MPC inhibitors increase lactate release by hepatocytes but do not affect expression of genes encoding gluconeogenic or lipogenic enzymes in DIO mice. (A) Total lactate pool size in media of hepatocytes treated with the indicated compounds as measured by mass spectrometry after three hours. Data presented as mean + standard error of the mean. n=3 *p<0.05 versus vehicle control. (B) The expression of the indicated genes in DIO mice treated for 3 days with the indicated drugs is shown. n=5 mice per group.
2

## Slide 3
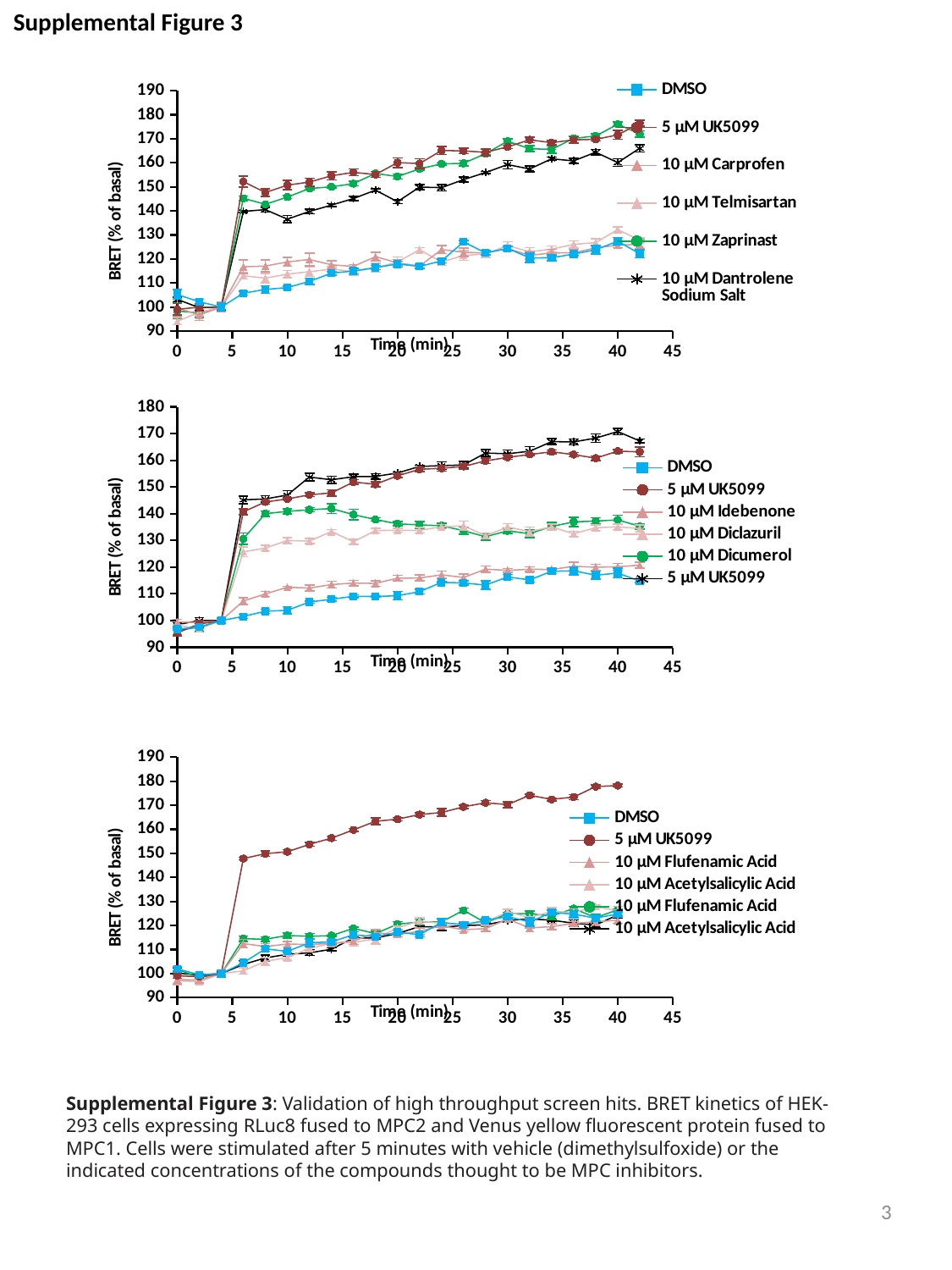

Supplemental Figure 3
### Chart
| Category | DMSO | 5 µM UK5099 | 10 µM Carprofen | 10 µM Telmisartan | 10 µM Zaprinast | 10 µM Dantrolene Sodium Salt |
|---|---|---|---|---|---|---|
### Chart
| Category | DMSO | 5 µM UK5099 | 10 µM Idebenone | 10 µM Diclazuril | 10 µM Dicumerol | 5 µM UK5099 |
|---|---|---|---|---|---|---|
### Chart
| Category | DMSO | 5 µM UK5099 | 10 µM Flufenamic Acid | 10 µM Acetylsalicylic Acid | 10 µM Flufenamic Acid | 10 µM Acetylsalicylic Acid |
|---|---|---|---|---|---|---|Supplemental Figure 3: Validation of high throughput screen hits. BRET kinetics of HEK-293 cells expressing RLuc8 fused to MPC2 and Venus yellow fluorescent protein fused to MPC1. Cells were stimulated after 5 minutes with vehicle (dimethylsulfoxide) or the indicated concentrations of the compounds thought to be MPC inhibitors.
3

## Slide 4
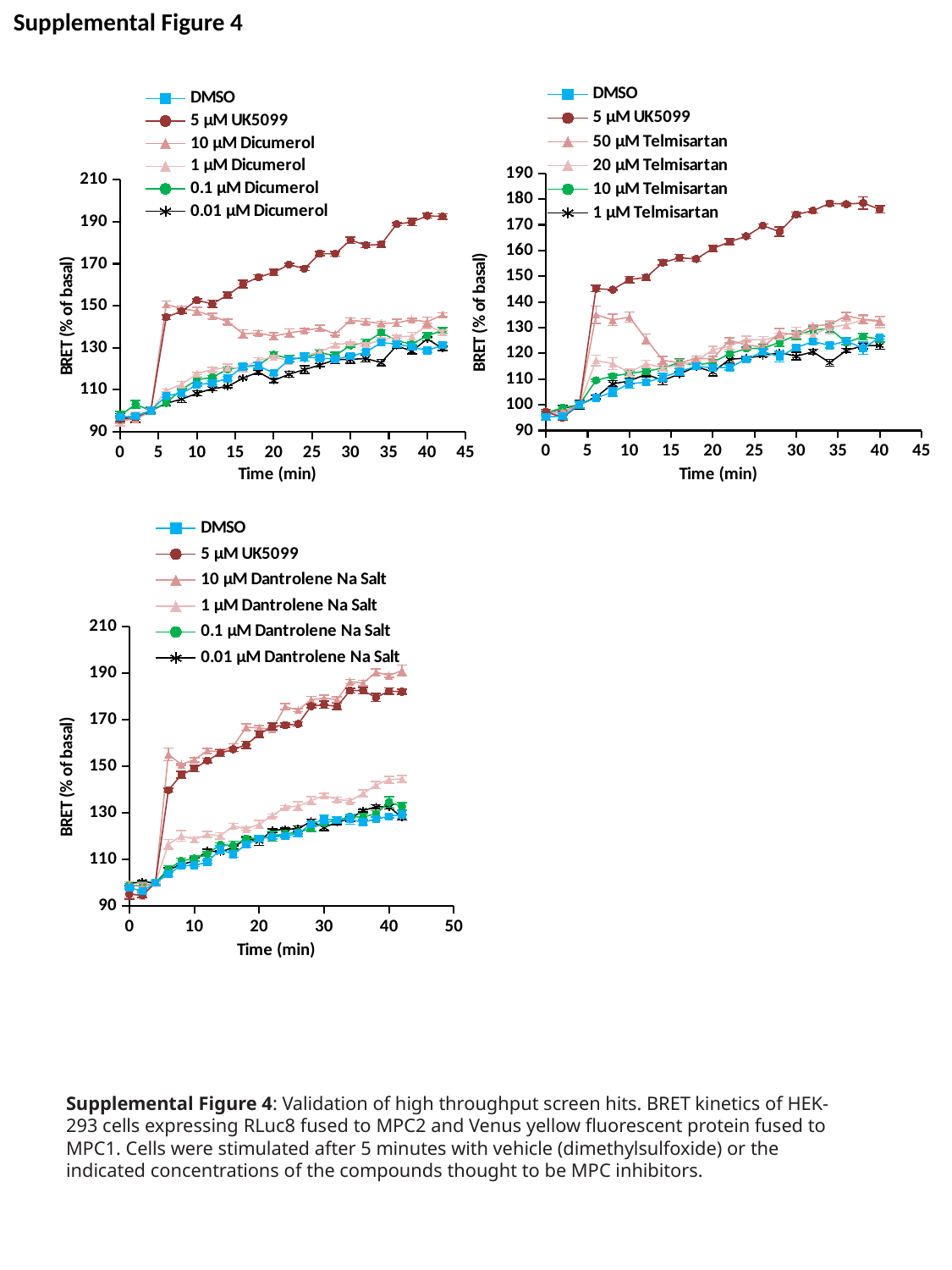

Supplemental Figure 4
### Chart
| Category | DMSO | 5 µM UK5099 | 50 µM Telmisartan | 20 µM Telmisartan | 10 µM Telmisartan | 1 µM Telmisartan |
|---|---|---|---|---|---|---|
### Chart
| Category | DMSO | 5 µM UK5099 | 10 µM Dicumerol | 1 µM Dicumerol | 0.1 µM Dicumerol | 0.01 µM Dicumerol |
|---|---|---|---|---|---|---|
### Chart
| Category | DMSO | 5 µM UK5099 | 10 µM Dantrolene Na Salt | 1 µM Dantrolene Na Salt | 0.1 µM Dantrolene Na Salt | 0.01 µM Dantrolene Na Salt |
|---|---|---|---|---|---|---|Supplemental Figure 4: Validation of high throughput screen hits. BRET kinetics of HEK-293 cells expressing RLuc8 fused to MPC2 and Venus yellow fluorescent protein fused to MPC1. Cells were stimulated after 5 minutes with vehicle (dimethylsulfoxide) or the indicated concentrations of the compounds thought to be MPC inhibitors.
